# Supplementary material for: Stable co-existence of Citrobacter rodentium with a lytic bacteriophage during in vivo murine infection
Source: mBio. 2025 Dec 29;17(2):e01944-25. doi: 10.1128/mbio.01944-25 (PMC12892995; doi:10.1128/mbio.01944-25)
Supplement: Supplemental Figures — Figures S1 to S5. [file mbio.01944-25-s0001.pdf]

Supplementary figures

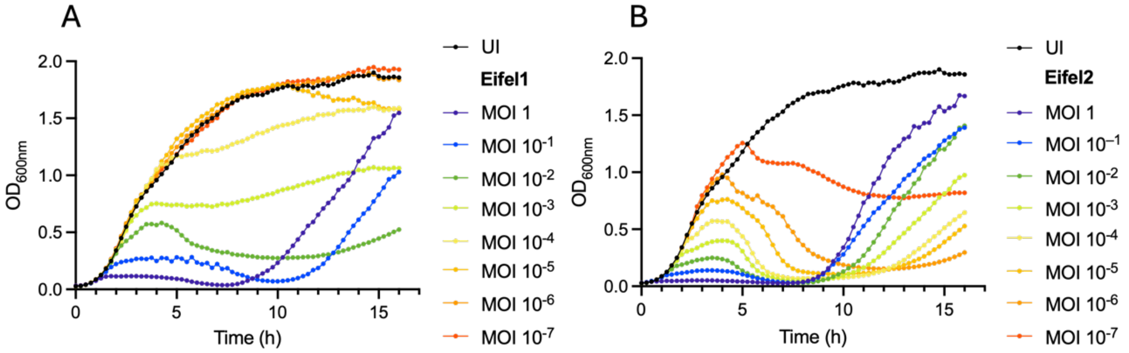

Figure S1. Lysis kinetics of CR_{WT} cultures infected with Eifel1 or Eifel2. (A-B) Lysis kinetics of CR_{WT} cultures infected with Eifel1 (A) or Eifel2 (B) at different MOIs or UI. The OD_{600} was measured every 15 min for 16 h. Data are shown as mean of n = 3 independent biological repeats.

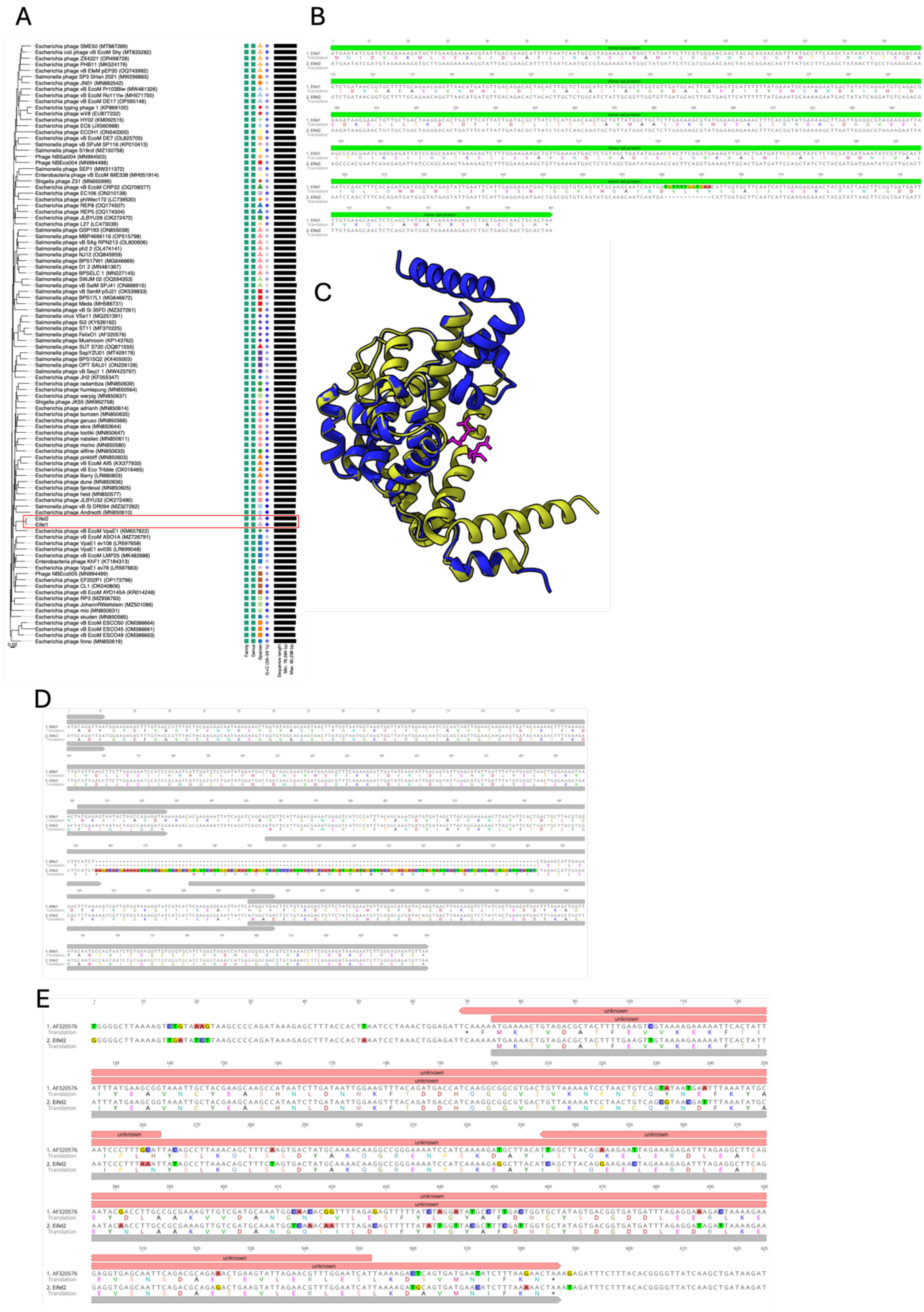

Figure S2. Comparative analysis of Eifel1 and Eifel2 genomic and protein features. (A) Genome-BLAST distance phylogenetic tree constructed by VICTOR (D6 formula) using the exemplar representative genomes of all *Felixounavirus* species. Annotations indicate

family, genus and species classification according to VICTOR. Combinations of colours and shapes represent new species. Eifel1 and Eifel2 are classified into a new distinct species. Additional annotations include GC content and genome length. (B) Sequence alignment of the minor tail protein of Eifel1 and Eifel2 and the Eifel1 minor tail protein aa translation. Deleted basebares in Eifel2 are highlighted in colour. (C) Schematic representation of the structural superposition of minor tail protein predictions for Eifel1 (yellow) and Eifel2 (blue), highlighting the deleted region (magenta). Structures were predicted using Alphafold 3. (D) Sequence alignment of the genomic region where an additional hypothetical gene is present in Eifel2 but absent in Eifel1, and its aa translation. (E) Sequence alignment of the additional gene present in Eifel2 with a homologous gene of *Salmonella* phage FelixO1 (accession number: AF320576), and their aa translations.

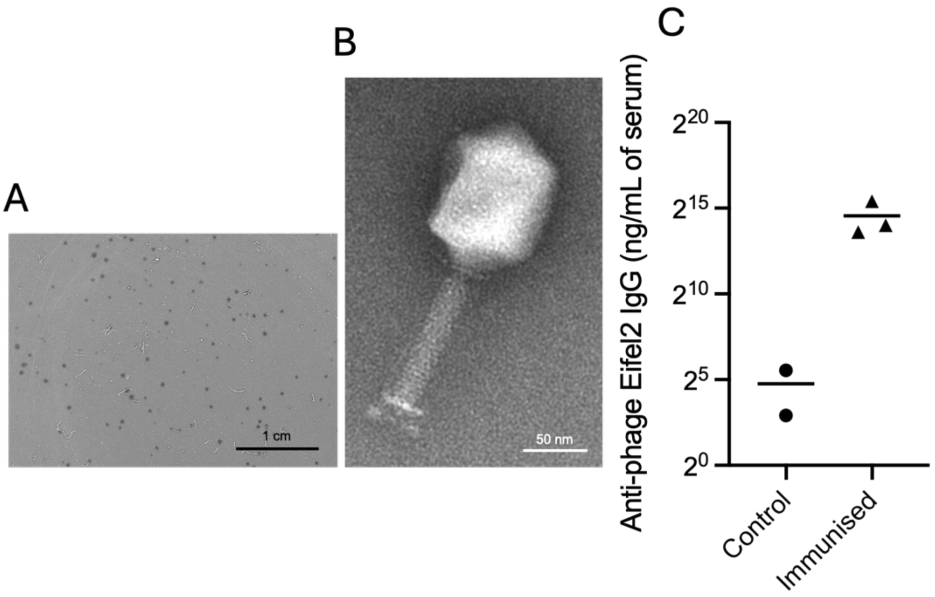

Figure S3. Eifel2 mouse immunisation and morphology of phage ColRes. (A) Plaque morphology of phage ColRes on a DLA plate with CR_{WT}. Scale bar: 1 cm. (B) Transmission electron micrographs of uranyl acetate negatively stained ColRes virions. Scale bar: 50 nm. (C) Anti-phage Eifel2 IgG levels in sera of immunised and mock-immunised mice, measured by ELISA. Each data point represents IgG levels in an individual mouse and bars indicate group means.

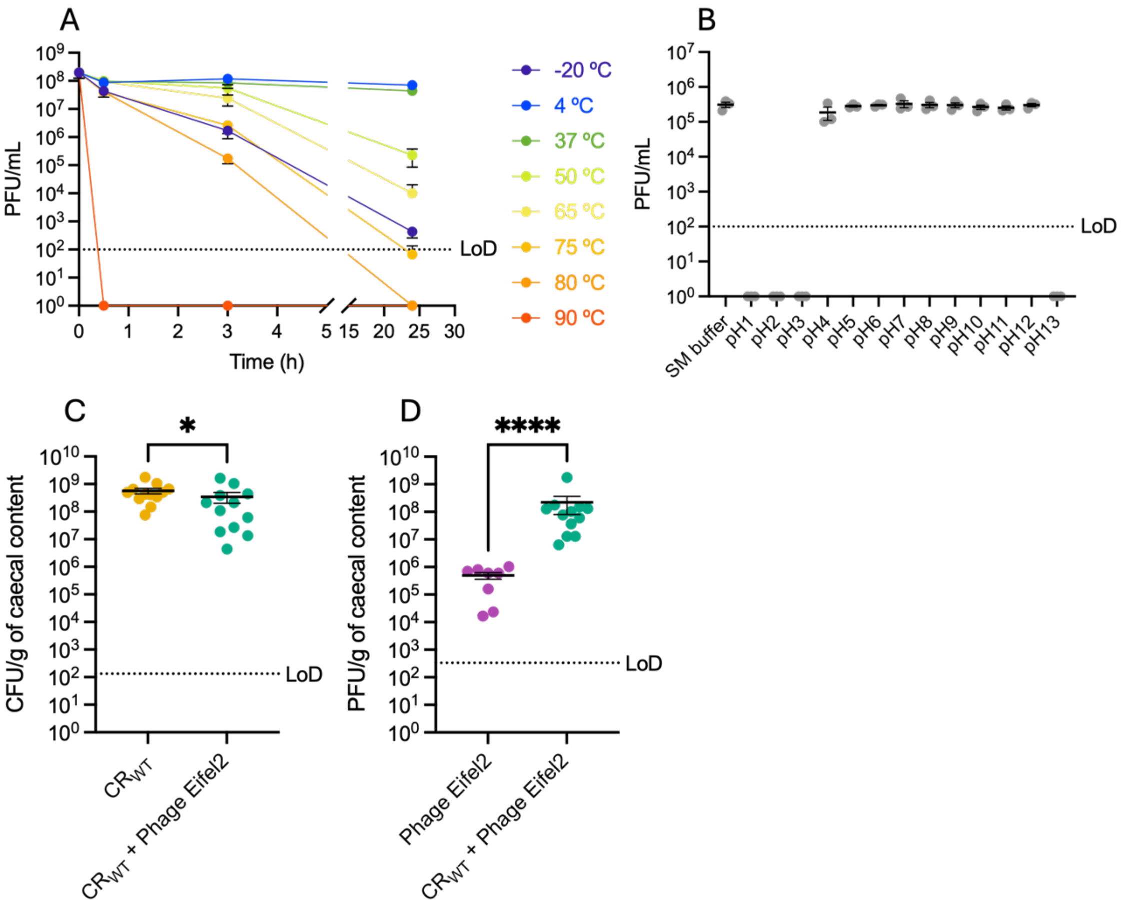

Figure S4. Characterisation of Eifel2 *in vitro* stability and *in vivo* activity. (A) Temperature stability of Eifel2 over 24 h of incubation at indicated temperatures, determined by DLA spot assays after 30 min, 3 h and 24 h. Data are shown as mean ± SEM from n = 3 independent biological repeats. (B) pH stability of Eifel2 after 24 h of incubation at indicated pH values, determined by DLA spot assays. Each dot represents PFU counts from one of n = 3 independent biological replicates and bars represent mean ± SEM. Statistical analysis of PFU counts above the LoD was performed using one-way ANOVA, with Dunnett's multiple comparison test. (C) Quantification of CR_{WT} in cecal contents at 8 dpi, expressed as CFU per g of cecal content, in infected mice receiving phage treatment or mock treatment. Sample sizes were n = 12 per group pooled from 3 independent experiments. (D) Quantification of Eifel2 in cecal contents at 8 dpi, expressed as PFU per g of cecal content, in infected or mock-infected mice receiving

phage treatment. Sample sizes were n = 8 (UI) and n = 12 (infected) pooled from 2 and 3 independent experiments, respectively. (A, B) Values below the LoD were set to 1 for plotting. (C, D) Each dot represents values from an individual mouse and lines represent mean ± SEM. Statistical analysis was performed using a Mann-Whitney test. (B, C, D) Statistical significance is indicated as: $P < 0.05$ (*); $P < 0.01$ (**); $P < 0.001$ (***); $P < 0.0001$ (****); non-significant comparisons are not shown.

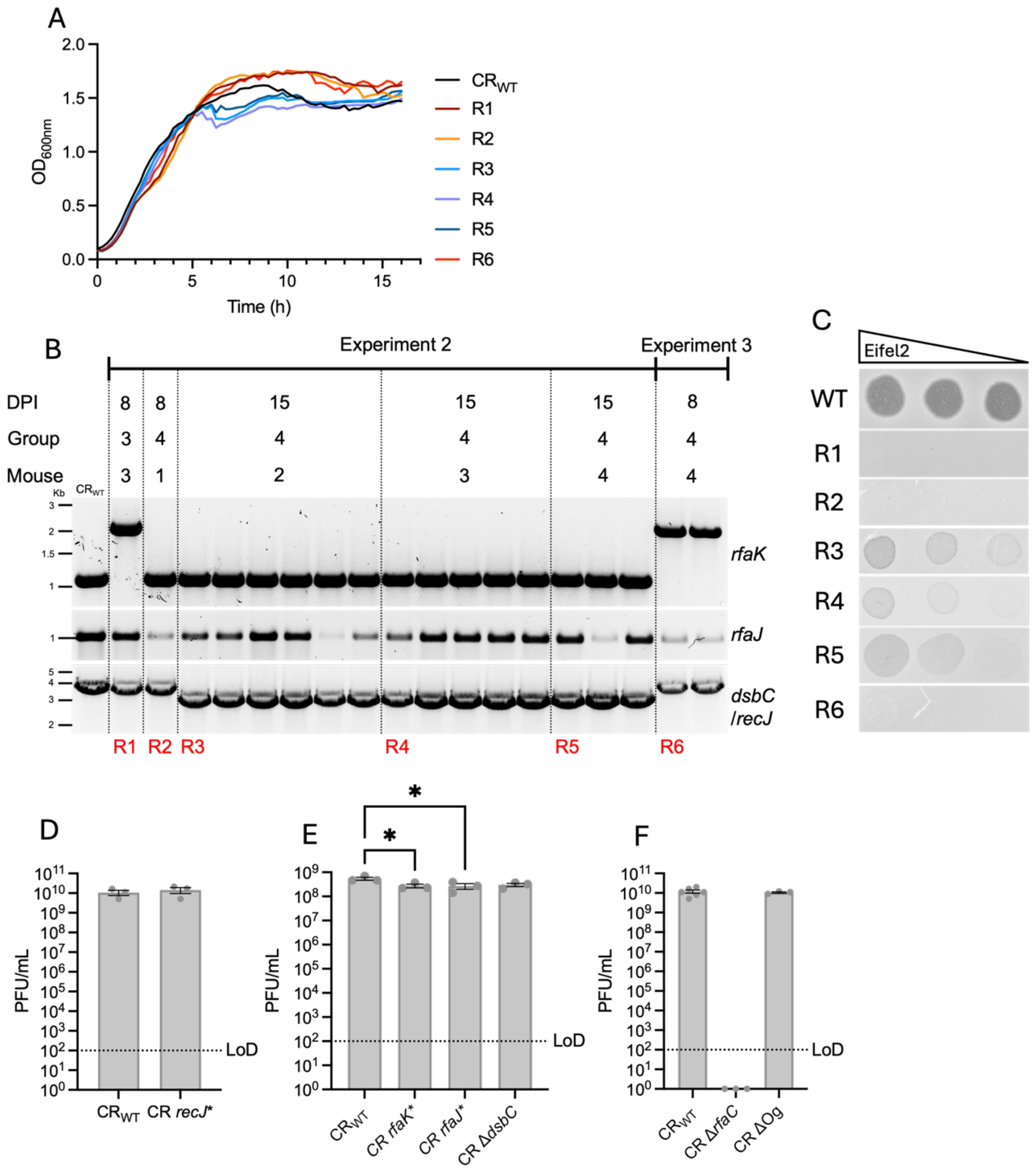

Figure S5. Additional characterisation of CR Eifel2-resistance phenotypes. (A) Growth curves of CR_{WT} and sequenced resistant fecal isolates (R1-R6) cultures in LB medium. OD_{600} was measured every 15 min for 16 h. Data are shown as mean of n = 3 independent biological repeats. (B) Agarose gel electrophoresis of PCR-amplified fragments of *rfaK*, *rfaJ* and *dsbC*/*recJ* loci from CR_{WT} and resistant fecal isolates (R1-R6). The expected product sizes for CR_{WT} fragments are: rfaK, 1149 bp; *rfaJ*, 1017 bp; *dsbC*/*recJ*, 3820 bp. Lane annotations indicate dpi, experimental replicate, experimental

group, mouse identifier and sequenced isolates names. (C) Representative images of DLA spot assays using Eifel2 (~10^{10} to ~10^{8} PFU/mL) on CR_{WT} and resistant fecal isolates (R1-R6). Images are representative of n = 3 independent biological replicates. (D-F) PFU counts from DLA spot assays of Eifel2 (D, F) and ColRes (E) on CR mutant strains (D, E) or Δ*rfaC* and ΔOg CR mutant strains (F). Data are shown as mean ± (SEM) of n = 3 independent biological replicates and each dot represents PFU counts for a biological replicate. Values below the LoD were set to 1 for plotting. Statistical analysis of strains with PFU counts above the LoD was performed using Welsh's *t* test or one-way ANOVA with Dunnett's multiple comparisons test. Statistical significance is indicated as: $P < 0.05$ (*); $P < 0.01$ (**); $P < 0.001$ (***); $P < 0.0001$ (****); non-significant comparisons are not shown.

Supplementary references

1. Bradshaw, J. *et al.* The exopolysaccharide Poly-N-Acetyl-Glucosamine (PNAG) coats Klebsiella pneumoniae in vivo. 2024.09.23.614408 Preprint at https://doi.org/10.1101/2024.09.23.614408 (2024).

2. Carson, D. *et al.* Citrobacter rodentium induces rapid and unique metabolic and inflammatory responses in mice suffering from severe disease. *Cellular Microbiology* **22**, e13126 (2020).

3. Chen, Y. *et al.* Draft Genome Sequences of Citrobacter freundii and Citrobacter murliniae Strains Isolated from the Feces of Preterm Infants. *Microbiol Resour Announc* **8**, e00494-19 (2019).

4. Choi, K.-H. *et al.* A Tn7-based broad-range bacterial cloning and expression system. *Nat Methods* **2**, 443–448 (2005).

5. Darfeuille-Michaud, A. *et al.* Presence of adherent Escherichia coli strains in ileal mucosa of patients with Crohn's disease. *Gastroenterology* **115**, 1405–1413 (1998).

6. Fey, P. D. *et al.* A Genetic Resource for Rapid and Comprehensive Phenotype Screening of Nonessential Staphylococcus aureus Genes. *mBio* **4**, e00537-12 (2013).

7. Figurski, D. H. & Helinski, D. R. Replication of an origin-containing derivative of plasmid RK2 dependent on a plasmid function provided in trans. *Proceedings of the National Academy of Sciences* **76**, 1648–1652 (1979).

8. Guzman, L. M., Belin, D., Carson, M. J. & Beckwith, J. Tight regulation, modulation, and high-level expression by vectors containing the arabinose PBAD promoter. *J Bacteriol* **177**, 4121–4130 (1995).

9. Herrero, M., de Lorenzo, V. & Timmis, K. N. Transposon vectors containing non-antibiotic resistance selection markers for cloning and stable chromosomal insertion of foreign genes in gram-negative bacteria. *Journal of Bacteriology* **172**, 6557–6567 (1990).

10. Hoiseth, S. K. & Stocker, B. a. D. Aromatic-dependent Salmonella typhimurium are non-virulent and effective as live vaccines. *Nature* **291**, 238–239 (1981).

11. Levine, M. *et al.* ESCHERICHIA COLI STRAINS THAT CAUSE DIARRHŒA BUT DO NOT PRODUCE HEAT-LABILE OR HEAT-STABLE ENTEROTOXINS AND ARE NON-INVASIVE. *The Lancet* **311**, 1119–1122 (1978).

12. Maher, M. M., Jordan, K. N., Upton, M. E. & Coffey, A. Growth and survival of E. coli O157:H7 during the manufacture and ripening of a smear-ripened cheese produced from raw milk. *Journal of Applied Microbiology* **90**, 201–207 (2001).

13. Mullineaux-Sanders, C. *et al.* Citrobacter amalonaticus Inhibits the Growth of Citrobacter rodentium in the Gut Lumen. *mBio* **12**, e02410-21 (2021).

14. Mulvey, M. A., Schilling, J. D. & Hultgren, S. J. Establishment of a Persistent Escherichia coli Reservoir during the Acute Phase of a Bladder Infection. *Infection and Immunity* **69**, 4572–4579 (2001).

15. Ruano-Gallego, D., Álvarez, B. & Fernández, L. Á. Engineering the Controlled Assembly of Filamentous Injectisomes in E. coli K-12 for Protein Translocation into Mammalian Cells. *ACS Synth Biol* **4**, 1030–1041 (2015).

16. Ruano-Gallego, D. *et al.* Type III secretion system effectors form robust and flexible intracellular virulence networks. *Science* **371**, eabc9531 (2021).

17. Schauer, D. B. & Falkow, S. Attaching and effacing locus of a Citrobacter freundii biotype that causes transmissible murine colonic hyperplasia. *Infection and Immunity* **61**, 2486–2492 (1993).

18. Watson, J. L. *et al.* Shigella sonnei O-Antigen Inhibits Internalization, Vacuole Escape, and Inflammasome Activation. *mBio* **10**, e02654-19 (2019).

19. Wong, J. L. C. *et al.* OmpK36-mediated Carbapenem resistance attenuates ST258 Klebsiella pneumoniae in vivo. *Nat Commun* **10**, 3957 (2019).

20. Wong JLC, Sanchez-Garrido J, Rattle J, Bradshaw J, Mishra V, Frankel G. 2025. citrOgen: a synthesis-free polysaccharide and protein antigen-presentation to antibody-induction platform. Nat Commun 16:8886.
